# xMAP Technology: Applications in Detection of Pathogens

**DOI:** 10.3389/fmicb.2017.00055

**Published:** 2017-01-25

**Authors:** Nikol Reslova, Veronika Michna, Martin Kasny, Pavel Mikel, Petr Kralik

**Affiliations:** ^1^Department of Food and Feed Safety, Veterinary Research InstituteBrno, Czechia; ^2^Department of Botany and Zoology, Faculty of Science, Masaryk UniversityBrno, Czechia; ^3^Department of Experimental Biology, Faculty of Science, Masaryk UniversityBrno, Czechia

**Keywords:** xMAP, magnetic microspheres, multiplex detection, nucleic acid detection, immunoassay, pathogen identification, diagnostics

## Abstract

xMAP technology is applicable for high-throughput, multiplex and simultaneous detection of different analytes within a single complex sample. xMAP multiplex assays are currently available in various nucleic acid and immunoassay formats, enabling simultaneous detection and typing of pathogenic viruses, bacteria, parasites and fungi and also antigen or antibody interception. As an open architecture platform, the xMAP technology is beneficial to end users and therefore it is used in various pharmaceutical, clinical and research laboratories. The main aim of this review is to summarize the latest findings and applications in the field of pathogen detection using microsphere-based multiplex assays.

## Introduction

High-throughput multiplex detection techniques are designed for the rapid, sensitive and specific testing of large numbers of analytes (nucleic acid assays, immunoassays, enzyme assays, or receptor-ligands) in a single biological sample. These techniques enable analysis of large numbers of samples. On the other hand, there are also classical single reaction detection methods based on determination of nucleic acids such as polymerase chain reaction (PCR) (Dunbar, 2006; Taylor et al., 2001), quantitative real-time PCR (qPCR) (Wuyts et al., 2015; Iannone et al., 2000), reverse transcription PCR (RT-PCR) (Weis et al., 1992) and reverse transcription quantitative PCR (RT-qPCR) (Bustin, 2000), or antibody-based tests like enzyme-linked immunosorbent assays (ELISA) (Engvall and Perlmann, 1971; Vanweeme and Schuurs, 1971) represent nowadays the “gold diagnostic standard” in many laboratories. Despite the previous implementation of these methods for routine rapid, sensitive, specific and cost-effective molecular diagnostics, their ability to simultaneously detect multiple analytes in a single reaction is limited and this limitation has yet to be overcome. The increasing amount of proteomic, transcriptomic and genomic sequence data from a large number of organisms accessible in public databases represents an exceptional opportunity for the development of new, multiplex detection technologies. The Luminex^®^ xMAP technology (x = analyte, MAP = Multi-Analyte Profiling) that was invented in the late 1990s represents such a platform that can benefit from all the advances in DNA research (Angeloni et al., 2014). Although PCR allows multiplex amplification of several targets in a single run xMAP as a methodology represents a significant step forward, and was designed with the aim of creating a high-throughput bioassay platform, enabling rapid, cost-effective, and simultaneous analysis of multiple analytes within a single biological sample. As an open architecture platform, the xMAP system holds many benefits for the end user and therefore it is used in pharmaceutical, clinical and research laboratories (Dunbar and Li, 2010). The main aim of this review is to summarize the state-of-the-art of xMAP technology applications in the detection of viral, bacterial, parasitical and fungal pathogens from different matrices.

## xMap Technology – in the Beginning were the Microspheres

**FIGURE 1:**
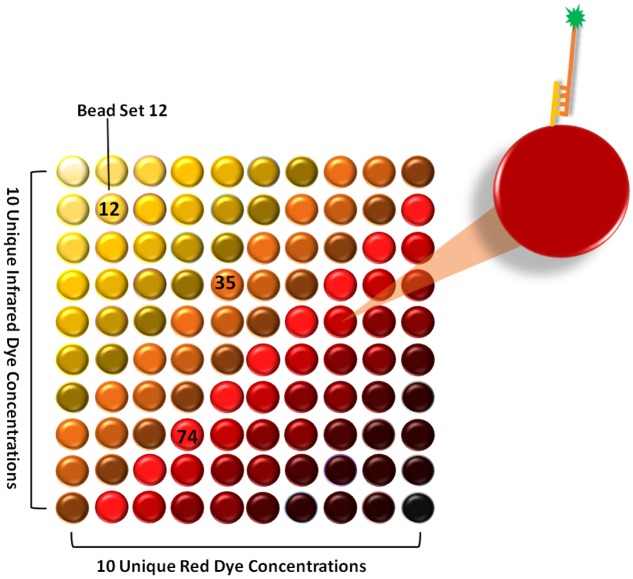
**The xMAP Technology based on internally dyed microspheres.** Different concentrations of red and infrared fluorophores were used to create 100 distinct microsphere sets. Each set is able to conjugate to a specific target molecule (yellow and orange lines = nucleic acid; green star = fluorescent reporter).

The principle of xMAP technology is based on the concept of a liquid (suspension) array. In contrast to the conventional microarray technology where the identity of the analyte is characterized by its position on the glass slide, the xMAP technology uses different sets of microspheres in a liquid suspension as determiners of analyte specificity. Microsphere sets are internally dyed with two spectrally different fluorophores. The spectral signature is unique for each microsphere set and is determined by different concentrations of internal dyes, producing a 100-member array of spectrally distinct microsphere sets (**Figure 1**). Integration of a third internal dye has allowed the expansion of up to 500-member microsphere sets (Dunbar and Li, 2010). The surface of each microsphere set allows a simple chemical coupling of various reagents specific to a particular bioassay, such as nucleic acid assays, immunoassays, enzyme assays or receptor-ligand assays. A further fluorescent reporter (e.g., Streptavidin-R-phycoerythrin, Alexa 532, Cy3) is coupled to a target molecule, which allows its detection after specific capture on the microsphere surface.

**FIGURE 2:**
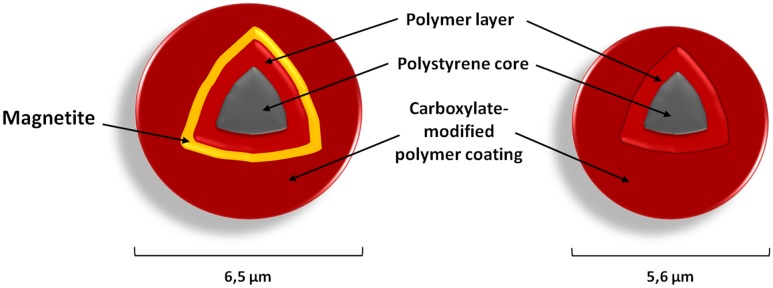
**Microsphere architecture.** The polystyrene divinylbenzene core is surrounded by a polymer layer, which is formed by polystyrene methacrylic acid (infusion of dyes). The surface of each microsphere is irregular, porous and carboxylated. Magnetic microspheres have an additional layer of magnetite within the polymer layer and so differ also in size.

There are different types of commercially available microspheres (**Table 1**), and their selection is generally determined by the type of instrumentation used for detection and the particular analyte of interest (Dunbar and Li, 2010; Houser, 2012). Basic microspheres are 5.6 μm polystyrene beads whose surface is covered by approximately 10^8^ carboxyl groups (COOH) for covalent coupling of capture reagents (Tang and Stratton, 2006). Magnetic microspheres (**Figure 2**) differ in size and structure through the addition of a magnetite layer (Dunbar and Li, 2010; Houser, 2012). Usage of magnetic beads improves washing efficiency as the magnetic separation step enables the elimination of unwanted sample constituents. Moreover, MagPlex-TAG microspheres are covalently pre-coupled with unique 24 base pair-(bp)-long anti-TAG oligonucleotides that serve as an anchor for target sequences containing the complementary TAG sequence. This proprietary TAG system (xTAG technology) is optimized to have minimum cross-reactivity. An assay can be easily designed by adding a complementary TAG sequence into the sequence of the primer or detection probe of interest and hybridization to the anti-TAG sequence on the microsphere surface.

**Table 1:** Commercially available microspheres.

Microsphere type	Size (μm)	Structure	Sets available	Instrument suitability	Analyte
MicroPlex^®^	5.6	Non-magnetic	100	Flow cytometry-based	All
MagPlex^®^	6.5	Magnetic	500	All xMAP	All
MagPlex-TAG^TM^	6.5	Magnetic	150	All xMAP	Nucleic acid
LumAvidin^®^	5.6	Non-magnetic	100	Flow cytometry-based	Proteins
SeroMAP^TM^	5.6	Non-magnetic	100	Flow cytometry-based	Proteins


### Mechanism of Signal Detection and Overview of Available Instruments

The analysis of beads is in general performed by two lasers. The red classification laser/LED (635 nm) excites the inner fluorescent dyes of the microspheres, thus identifying a specific microsphere set according to its spectral signature. If the analyte of interest is present, the green reporter laser/LED (525–532 nm) recognizes the fluorescent reporter bound to the captured analyte on the microsphere surface.

There are approximately 10^4^ microspheres from each set present in a single sample. This number represents the range in xMAP, in which it is possible to perform determination of quantity according to a calibration curve, similarly to qPCR. However, one must bear in mind that inclusion of a PCR amplification step prior to xMAP analysis does not reveal the real number of DNA molecules present in the original sample, but can only be used for the approximate estimation of DNA quantity. Therefore, xMAP can provide only semi-quantitative data.

The simultaneous reading of both spectra is performed in purpose-designed readers (**Table 2**). They differ by their mechanisms of fluorescence capture and by the maximum number of samples that can be analyzed.

**Table 2:** Detection instruments compatible with xMAP technology.

Instrument	Compatibility	Strategy	Analytes/reaction	Microplate type
Luminex MAGPIX^®^	Magnetic microspheres	Immobilization of microspheres in magnetic field	50	96-well plate
Luminex100^®^/200^TM^	All types of microspheres	Flow cytometry-based	100 (80 with MagPlex)	96-well plate
FlexMAP 3D^®^	All types of microspheres	Flow cytometry-based	500	96 and 384-well plate


**FIGURE 3:**
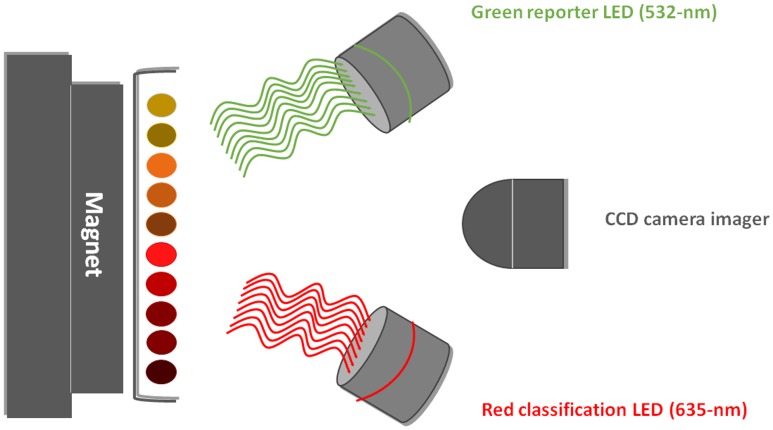
**Principle of analysis by the MAGPIX fluorescent imager.** Magnetic microspheres immobilized on a magnet are recognized by LEDs and a CCD camera records the picture (LED, light-emitting diode; CCD, charge-coupled device).

The basic detection instrument, which is called MAGPIX, is compatible only with magnetic microspheres (MagPlex and MagPlex-TAG). The principle of microsphere analysis in the MAGPIX instrument is based on their immobilization in the monolayer on the magnetic surface (**Figure 3**). Unlike flow-based instruments, the fluorescent imager of the MAGPIX system reads all the microspheres at once, while generating data that is comparable with other methods. Reading a 96-well-plate takes about 60 min. The maximal reading capacity of MAGPIX instruments is limited to 50 bead sets.

Advanced detection instruments – the Luminex 100/200 (Bio-Plex 200) and FlexMAP (Bio-Plex) 3D – are based on flow cytometry principles. The microspheres with bound analyte are focused into a rapidly flowing fluid stream. Each microsphere is then individually detected and digitally processed as the stream passes through the imaging cuvette. Flow cytometry-based platforms are convenient for applications with samples of limited size. The reading of a 96-well-plate is faster than in the MAGPIX system and takes 45 min or less. The capacity of the 3D platform is further increased by the possibility of analyzing 384-well plates.

## Microsphere-Based Multiplex Assay Formats

The microsphere-based technology can be applied in various assay formats, which can be divided, according to the type of analyte, into microsphere-based multiplex nucleic acid assay formats (MBMNA) and microsphere-based multiplex immunoassays (MBMI).

In general, xMAP-based assay formats are in comparison to other commonly used methods very open and flexible, ensuring the result data within few hours, while requiring only minimal amounts of sample.

Detection assays based on nucleic acids have a potential for high levels of multiplexing, approaching the levels of sensitivity achieved by target amplification methods like multiplex PCR or TaqMan chemistry assays, while using the same protocols of DNA/RNA extraction. Multiplex oligonucleotide ligation PCR assay format (MOL-PCR) is able to simultaneously perform detection and identification, strain typing, detect antibiotic resistance determination, virulence prediction, etc., thereby surpasses other methods like Multiplex Ligation-dependent Probe Amplification (MLPA) or qPCR. The disadvantage of technology is that it is not capable to perform quantitative analysis like qPCR, because providing only semi-quantitative data.

xMAP immunoassays surpass the common enzyme immunoassays in the ability of multiple simultaneous detection, while requiring smaller amount of sample and lower cost. Moreover, these assay formats produce superior dynamic range and sensitivity.

### Nucleic Acid Assays (MBMNA)

xMAP technology is applicable in numerous nucleic acid assay formats such as, e.g., gene expression analysis, microRNA analysis, single nucleotide polymorphism (SNP) analysis or specific sequence detection. Basically, nucleic acid assays can be developed by coupling sequence-specific capture oligos to magnetic microspheres or by use of xTAG technology (Angeloni et al., 2014).

When performing xMAP analysis of nucleic acids it is essential to include PCR amplification to enrich the number of targets in the sample to detectable levels. There are two general strategies for including a PCR step in the detection of pathogens using xMAP technology. The main difference between the two lies in which phase the PCR amplification is applied. In direct DNA hybridization (DDH), allele-specific primer extension (ASPE), single base chain extension (SBCE), and Oligonucleotide ligation assay (OLA) all the target DNA sequences are amplified in multiplex PCR prior to hybridization to microspheres. The disadvantage of these methods is that in assays containing large amounts of targets multiplex PCR leads to amplification bias, which is caused by the different lengths of the amplicons (Nolan et al., 2001). In contrast, in the multiplex oligonucleotide ligation PCR assay (MOL-PCR) sequence discrimination by detection probes occurs before the amplification step, which can subsequently be run just in singleplex PCR with universal primers.

#### Direct DNA Hybridization (DDH)

**FIGURE 4:**
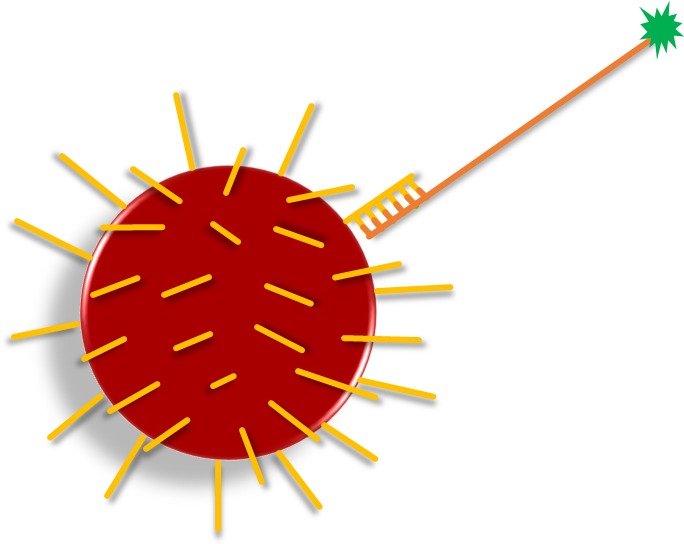
**Direct DNA hybridization (DDH, yellow lines = capture oligonucleotide; orange line = amplified target sequence; green star = fluorescent reporter).** Target DNA sequence is amplified, while one of the primers is fluorescently labeled. Amplicons are then specifically hybridized (according to complementarity) to capture oligonucleotides on the microsphere surface.

Direct DNA hybridization is one of the basic approaches used for the selective identification of sequences of interest from heterogeneous mixtures of DNAs (**Figure 4**). It is often used, e.g., for identification of species (Defoort et al., 2000; Page and Kurtzman, 2005; Righter et al., 2011; Liu Y. et al., 2012) or genotyping of pathogens (Letant et al., 2007; Zubach et al., 2012). In DDH, the amplification of target sequences is ensured by specific primer pairs, and one primer from each pair is fluorescently labeled at the 5′ end, permitting detection of the amplicon (Christopher-Hennings et al., 2013). The subsequent incubation of amplicon with microspheres leads to a direct and specific hybridization between matching capture and target sequences. Amplicon sequences should be 100–300 bp in length to minimize steric hindrance during hybridization and the capture sequence on microspheres should be 18–20 bp in size (Dunbar, 2006). The specificity of the capture sequences and stringency of hybridization conditions allow discrimination up to SNP. If the SNP or mutation discrimination is intended, the presumed mismatch should be located at the center of the capture sequence (Livshits and Mirzabekov, 1996). This assay format then requires a unique capture sequence coupled to a specific microsphere set to score each SNP allele (Kellar and Iannone, 2002).

#### Allele-Specific Primer Extension (ASPE)

**FIGURE 5:**
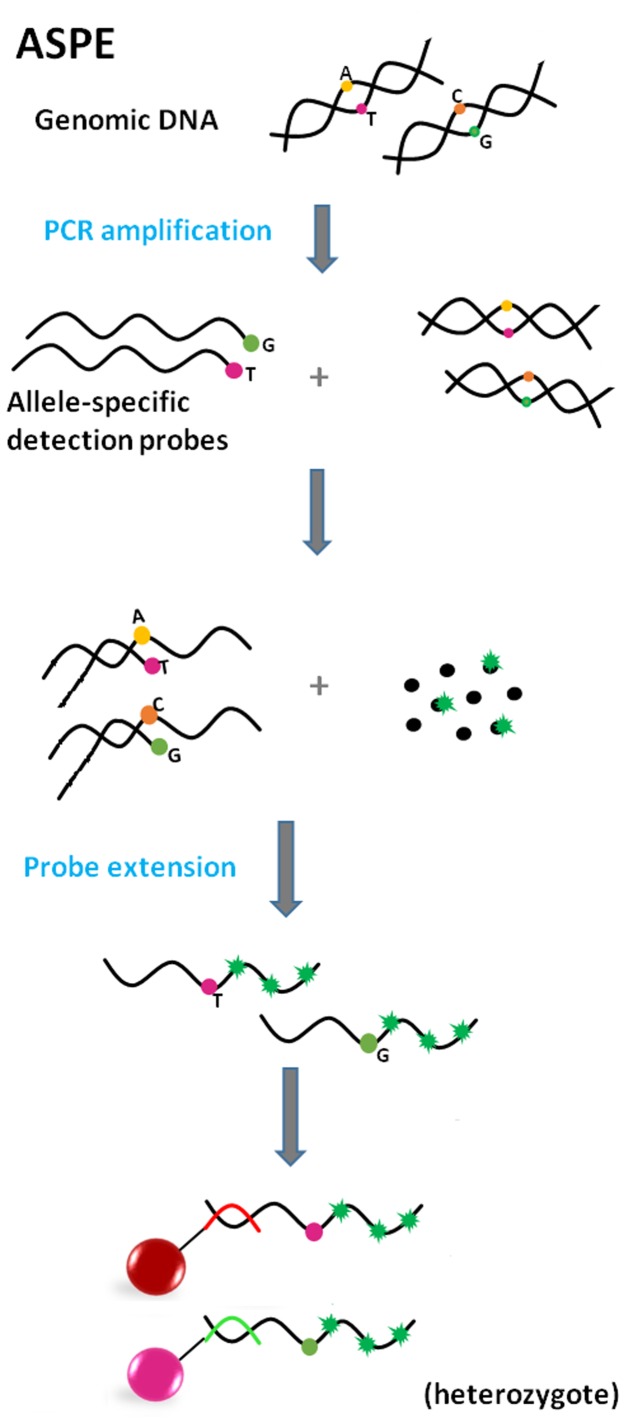
**Principle of Allele-specific primer extension (ASPE) (red and green line = anti-TAGs; green star = fluorescent reporter).** Allele-specific detection probes, differing in one nucleotide on the polymorphic side, hybridize to amplified target sequence. After addition of DNA polymerase and dNTPs (one of which is fluorescently labeled), molecules are extended according to complementarity. Products are captured by anti-TAGs on the specific microsphere set.

Allele-specific primer extension (**Figure 5**) is an approach usually used for determination of allelic variants of pathogens (Page and Kurtzman, 2005; Lin et al., 2008). The defining characteristic of ASPE is the extension of two allele-specific detection probes, which contain a polymorphic site at the 3′ end, defining the particular allele variant. In this arrangement, DNA polymerase can extend detection probes by incorporation of dNTPs (one nucleotide is labeled, e.g., biotin-dCTP), if the allele is present in the sample. Just one probe is extended in the case of a homozygous target; conversely, in heterozygotes both probes are extended. The fluorescence signal is generated by a fluorophore bound to labeled dNTPs, incorporated within the extended probe.

#### Single Base Chain Extension (SBCE)

**FIGURE 6:**
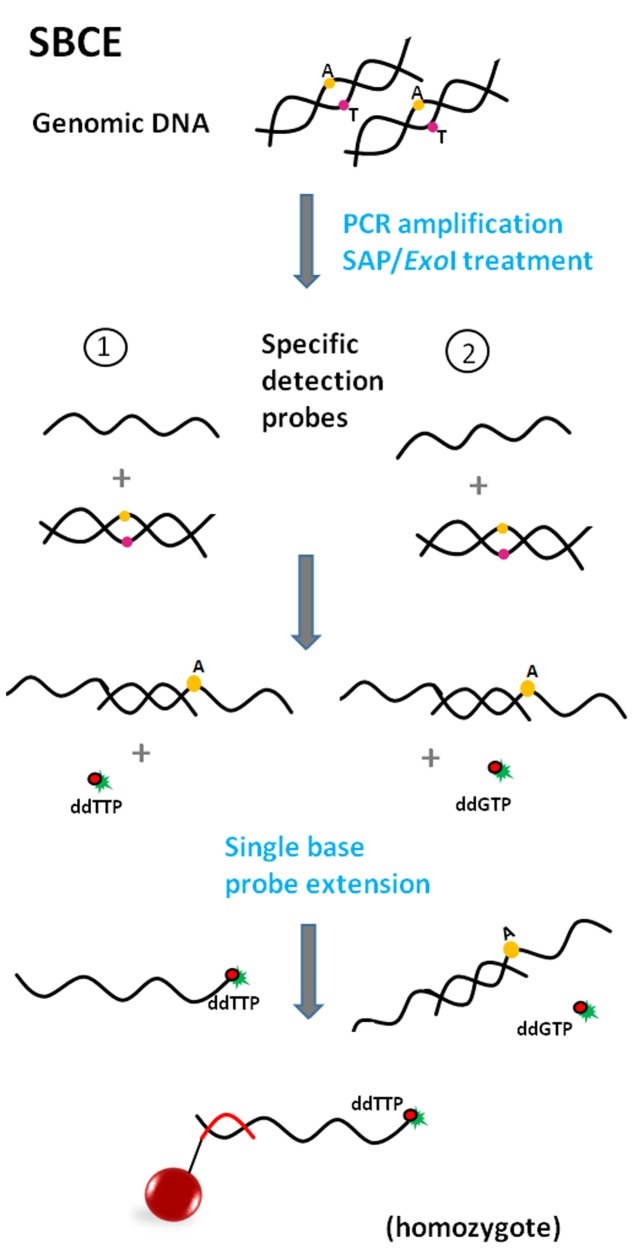
**Principle of Single base chain extension (SBCE) (red dot = dideoxynucleotide; green star = fluorescent reporter; red line = anti-TAG).** Specific detection probes are terminated one base before the polymorphic site. Utilization of fluorescently labeled dideoxynucleotides necessitates a separate reaction for each nucleotide in focus (minimally two). Target DNA hybridizes with probes after amplification but only the mix with the proper ddNTP leads ultimately to the synthesis of a labeled product, which is captured by anti-TAG on the microsphere surface.

The use (Taylor et al., 2001) and assay format of SBCE is similar to the previously described ASPE. However, there are slight differences, mainly in the design of detection probes. In the case of SBCE (**Figure 6**), probe sequences are terminated one base before the polymorphic site (Chen et al., 2000; Ye et al., 2001). Due to this design the labeled dideoxyribonucleoside triphosphate (ddNTP) terminators serve as a “query” nucleotide and are used for single base probe extension at the same time; this assay requires the setting up of separate reactions for each of the four ddNTPs (ddC, ddG, ddA, and ddT). Moreover, PCR products from the previous step of PCR amplification of the target sequence need to be treated with exonuclease I and shrimp alkaline phosphatase (*ExoI*/SAP) before use as a template in the SBCE reaction (Ye et al., 2001; Dunbar, 2006) to get rid of unincorporated primers and dNTPs. Although SBCE has been proven to be highly specific and reliable (Chen et al., 1999; Syvanen, 1999), it is in the process of being replaced by less laborious methods.

#### Oligonucleotide Ligation Assay (OLA)

Oligonucleotide ligation-based formats include a ligation step of two oligonucleotide detection probes, which occurs in the presence of a target sequence of a specific pathogen. These assays are based on the ability of detection probes to hybridize next to each other on a complementary target DNA sequence (Landegren et al., 1988). If there are no mismatches near the junction site and there is a phosphate group at the 5′ end of a second probe (necessary for phosphodiester bond formation), annealing occurs; DNA ligase then recognizes the nick and forms a covalent bond between adjoining nucleotides while creating a single-stranded DNA molecule (Iannone et al., 2000). The most crucial step during the multiplexing of different ligation assays is the design of suitable probes with similar melting temperatures of between 51 and 56°C (Dunbar, 2006).

**FIGURE 7:**
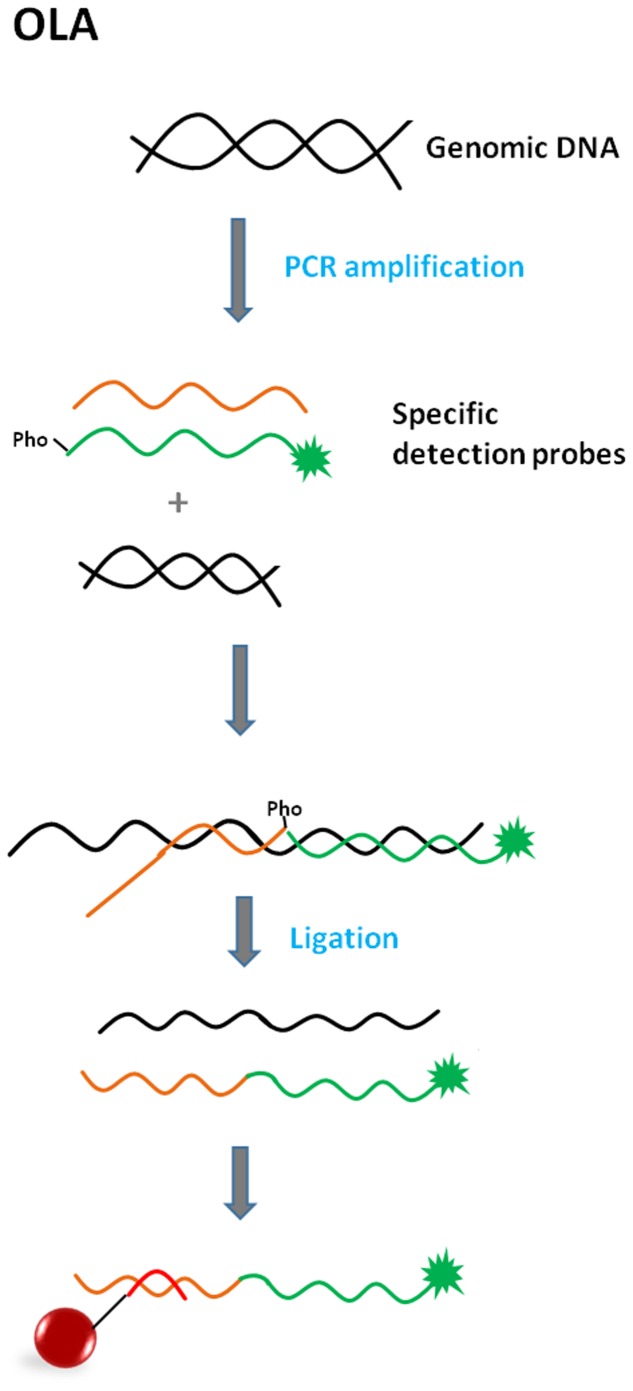
**Principle of Oligonucleotide ligation assay (OLA) (green star = fluorescent reporter; Pho = phosphate group; red line = anti-TAG).** The target DNA sequence is PCR-amplified prior to the ligation step of the annealed probes. One of the detection probes consists of a sequence complementary to the target sequence (polymorphic site at the 3′ end if SNP identification is needed) and also an additional TAG tail sequence. The second detection probe is fully complementary to the target sequence and serves as a reporter due to its fluorescent label at the 3′ end. Detection probes bind next to each other, DNA ligase recognizes the nick and makes a bond. The product is captured by anti-TAG on the microsphere surface.

In the OLA assay format, the target DNA sequence is PCR-amplified prior to the ligation step of the annealed probes (**Figure 7**). OLA is suitable for SNP genotyping (Iannone et al., 2000; Taylor et al., 2001; Ye et al., 2001).

#### Multiplex Oligonucleotide Ligation PCR Assay (MOL-PCR)

**FIGURE 8:**
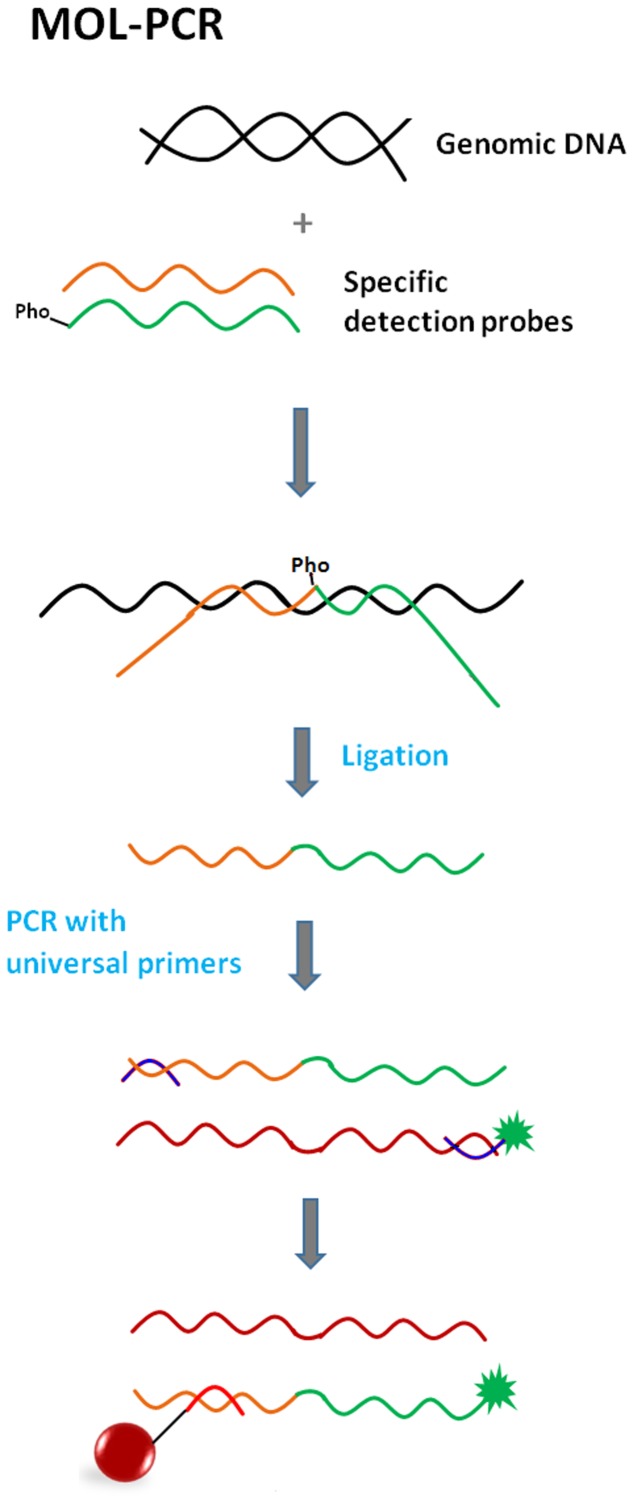
**Principle of Multiplex oligonucleotide ligation PCR assay (MOL-PCR) (orange line = detection probe 1; green line = detection probe 2; blue lines = universal PCR primers; burgundy line = amplified negative strand; green star = fluorescent reporter; Pho = phosphate group; red line = anti-TAG).** Specific detection probes bind next to each other to target sequence via complementary parts, while the parts including the TAG sequence and binding sites for PCR primers form tails sticking out into space. DNA ligase recognizes the nick and makes a bond. The complex sequence of ligated probes becomes a template for singleplex PCR with universal primers; one of the primers is fluorescently labeled. Labeled amplicon hybridizes via its TAG sequence to capture anti-TAG on the microsphere.

The multiplex oligonucleotide ligation PCR assay represents an improved version of the previous OLA assay format. One advantage is that ligation is carried out prior to the PCR-amplification (**Figure 8**) (Nolan and White, 2004). Unlike in the OLA assay, one of the detection probes consists of a sequence complementary to the target sequence and an extension composed of the TAG sequence and primer binding site. The second probe is the same as the first except for the absence of the TAG sequence. Each probe pair is specific for a particular target sequence, but all pairs share the same primer sequence. Basically, these modular detection probes anneal to a target sequence, ligate into a complex single-stranded DNA molecule and only if this occurs does the molecule become a template for singleplex PCR using a universal pair of primers (one is fluorescently labeled). Additionally, all the ligation products are very similar in length (approximately 100 bp -120 bp), so the use of a universal primer pair during PCR makes the simultaneous amplification of many short fragments highly feasible. All these facts ensure that MOL-PCR is not susceptible to the amplification bias that is characteristic of multiplex PCR or previously mentioned formats. Only a minimal amount of target/sample is required.

The MOL-PCR upgrade has the potential to have widespread impact on genomic assays, because not only is sequence detection and SNP identification possible, but the detection of indels (insertion/deletion), screening tests for pathogens (virus, bacteria, fungi) from various matrices or determination of antibiotic resistances is also feasible (Deshpande et al., 2010; Thierry et al., 2013; Wuyts et al., 2015). MOL-PCR could replace, e.g., MLPA or qPCR in certain applications in routine diagnostics (Deshpande et al., 2010).

### Microsphere-Based Multiplex Immunoassay (MBMI)

**FIGURE 9:**
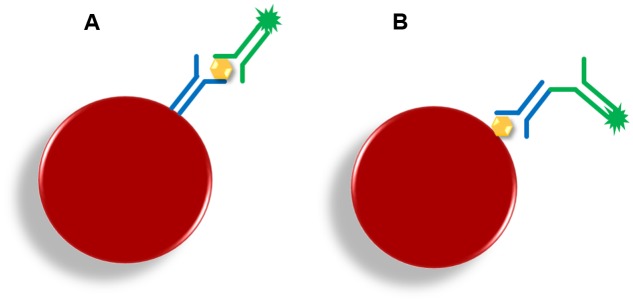
**Principle of microsphere-based multiplex immunoassays.** **(A)** Capture sandwich (CS; yellow hexagon = target; blue Y = capture antibodies; green Y = detection antibody; green star = fluorescent reporter); **(B)** Indirect serological assay (ISA; yellow hexagon = capture antigen; blue Y = specific target antibody; green Y = detection anti-antibody; green star = fluorescent reporter).

Microsphere-based multiplex immunoassay (MBMIs) are typically biochemical tests that allow the detection or measuring of the concentration of an analyte (protein) in a solution through the use of an antibody or immunoglobulin (Angeloni et al., 2014). Single-analyte ELISA cannot support simultaneous detection of multiple specific antibody responses within a single serum sample (Bokken et al., 2012), and has further disadvantages, such as the requirement for a relatively large amount of sample, negligible non-specific binding or increased background. MBMIs represent an alternative for commonly used indirect tests like ELISA. The conversion of an ELISA assay to the MBMI format is uncomplicated, efficient, cost-saving and produces an assay with superior dynamic range and sensitivity (Baker et al., 2012). MBMIs are often used in the diagnostics of various pathogens including multicellular organisms, such as e.g., parasites, in tests where the current methods are not sensitive enough. The methods of choice are usually Capture Sandwich (CS) and Indirect Serological Assay (ISA) (**Figure 9**). However, the problems typical for methods based on serology remain: the need for periodical testing in order to avoid false negative results resulting from a wide and inevitable lag between infection and development of a specific response against a parasite in the form of IgG antibodies (sero-positivity) (Nockler et al., 1995).

#### Capture Sandwich (CS)

The CS assay utilizes microspheres covalently coupled with a capture antibody (polyclonal antibodies should be purified and mono-specific) that takes up target molecules from the sample. This complex is recognized by a labeled detection antibody (Baker et al., 2012; Angeloni et al., 2014). The CS format can be used in cases where, for example, confirmation of pathogen identity within the inflammatory focus or altered tissue is needed.

#### Indirect Serological Assay (ISA)

In contrast to CS, in ISA a specific antibody against an antigen coupled with a microsphere is captured. If the binding of serum antibody to antigen occurs, a labeled secondary antibody (anti-antibody) then provides the visualization. ISA is typically used for serological screenings (monitoring and prevention purposes) that are carried out on serum samples (van der Wal et al., 2013).

## Applications of xMap Technology

The xMAP technology is used in many different applications. This chapter describes the use of this technology for multiplex detection of viral, bacterial, parasitical and fungal agents using the microsphere-based multiplex nucleic acid-assay formats (MBMNA) and microsphere-based multiplex immuno-assay formats (MBMI) described above.

### Multiplex Detection and Typing of Viruses

Viruses are a very diverse group of infectious agents and are divided into groups according to a number of properties, e.g., type of nucleic acid, the presence of the viral envelope, antigenic structure, mode of transmission, pathogenicity, etc. They can be classified also according to the syndromes which they cause and mode of transmission, e.g., respiratory viruses, viruses causing gastroenteritis, tumors, hepatitis, rashes or neuroviruses.

To date, the majority of applications that enable multiplex viral detection and identification are based on the capture of viral nucleic acid by adoption of various DDH modifications.

Respiratory viruses are causative agents of the most common diseases of the human upper and lower respiratory tract, which are often associated with significant patient morbidity and mortality (Berry et al., 2015), e.g., H5N1 subtype of highly pathogenic influenza A virus (Neumann et al., 2010).

The MBMNA method for more effective detection and genotyping of H5N1 viral isolates from clinical samples comprising pharyngeal swabs and tracheal aspirates was developed and its efficiency was compared with RT-PCR and qPCR (Zou et al., 2007). The results using the MBMNA approach showed that this assay is vulnerable to viral mutations although the primers were designed according to conserved sequences. Therefore, there is a need to monitor viral mutations in order to reduce false-negative results and add new primers and probes to adapt to the mutations, which is a disadvantage of MBMNA. On the other hand, MBMNA holds a number of advantages compared to RT-qPCR and qPCR, e.g., allele-specific probes with TAG sequences can be recognized by a universal set of primers, thus potentially eliminating the problem with different primer sets (which may be incompatible) used in conventional methods. Moreover, amplification is carried out with a single set of universal primers where only one primer is labeled; therefore, the background is low and no post-PCR cleanup is required.

Another application of the MBMNA method was developed for the identification of human adenoviruses (HAdVs). Conventional serological identification of HAdVs serotypes is a time consuming process. Target-specific extension (TSE), which is a variant of ASPE was suggested to accelerate identification through the use of MBMNA for simultaneous identification of different serotypes; this is not possible using commercially available neutralization tests, antibody studies, or antigen detection by immunofluorescence or conventional PCR (Washington et al., 2010). Universal primers were used for nonspecific PCR amplification and serotype-specific probes coupled to tags were used for TSE. This MBMNA procedure is methodically simple, the cost is relatively low, and it enables diagnosis of up to five HAdV serotypes in a single reaction.

Besides the in-house assays described above, commercial kits have also been developed for the detection of respiratory viruses by xMAP, e.g., xTAG^®^ Respiratory Viral Panel (xTAG RVP) (Krunic et al., 2011). xTAG RVP is multiplex nucleic acid test designed for detection of multiple respiratory virus nucleic acids in human nasopharyngeal swabs (Selvaraju and Selvarangan, 2012; Smith et al., 2012). Qualitative detection of a panel including respiratory syncytial virus (RSV), Influenza A virus (influenza A matrix, H1 subtype, H3 subtype, H5 subtype), Influenza B (Parainfluenza 1,2,3, and 4), Metapneumovirus (hMPV), HAdV, Entero-Rhinovirus, Corona NL63, Corona HKU1, Corona 229E, Corona OC43, and Bocavirus is possible. Bacteriophage MS2 and bacteriophage λ DNA were used as the internal controls. The detection of respiratory virus targets using the xTAG RVP, which detects 20 respiratory viral targets, was compared with individual qPCR nucleic acid amplification tests (NATs) (Pabbaraju et al., 2008). The xTAG RVP can detect all the respiratory viral targets included in the in-house NAT panel, which is used for detection of Influenza A, B viruses (IFVA, IFVB), parainfluenza virus types 1 to 4 (PIV 1–4), RSV, hMPV, and respiratory adenovirus types (ADV). Of the 1,530 samples tested, 532 were positive by xTAG RVP and 580 by in-house NATs for these targets. This gives the xTAG RVP a sensitivity of 91.2% and a specificity of 99.7%; in addition, xTAG RVP can detect picornaviruses (the in-house assays did not detect 88 picornaviruses) and coronaviruses and can subtype IFVA positives simultaneously. The xTAG RVP includes all the respiratory viral targets that are tested routinely for the diagnosis of acute respiratory tract infections; further, the technology is flexible and can easily allow for incorporation of other targets (e.g., human bocavirus) in the future.

The xTAG RVP assay was subsequently modified and was marketed as the xTAG RVP Fast assay, which has a simpler protocol; the results are obtained in a shorter time and handling of the amplified product is not required (amplified DNA is mixed with TAG primers specific to each viral target), which could be a potential contamination risk (Pabbaraju et al., 2011). The respiratory samples were tested for a variety of respiratory viral targets by xTAG RVP and xTAG RVP Fast in parallel. The xTAG RVP was more sensitive than xTAG RVP Fast (88.6% versus 77.5%) for all the viral targets; in addition, some targets (influenza B virus, parainfluenza virus type 2, and human coronavirus 229E) were not detected using xTAG RVP Fast and, e.g., the sensitivity for detection of IFVB was very low (41.3%). Therefore, it is not suitable as the primary assay for the detection of IFVB.

In addition to respiratory viral diseases the MBMNA was successfully applied also for detection of viral pathogens causing acute viral gastroenteritis. Acute viral gastroenteritis is usually caused by four distinct families of viruses: rotaviruses, noroviruses, astroviruses, and adenoviruses (Liu Y. et al., 2012). The authors focused on simultaneous detection of rotavirus A (RVA), noroviruses (NoVs), sapoviruses (SaV), human astrovirus (HAstV), enteric adenoviruses (EAds) and human bocavirus 2 (HBoV2). Altogether 140 fecal samples were tested using the MBMNA and RT-PCR in parallel. The specificity of MBMNA was equal to the conventional RT-PCR (>90%), but MBMNA was faster in terms of detection of different viral pathogens in one tube (Liu Y. et al., 2012). The studies of (Hamza et al., 2014) were also directed to the detection of human enteric viruses (human adenovirus (HAdV), human polyomavirus (HPyV), enterovirus (EV), rotavirus (RoV), norovirus GI (NoVGI), and norovirus GII (NoVGII), but environmental water samples were tested (Hamza et al., 2014). MBMNA provided high specificity and no cross-reactivity, but was not as sensitive as qPCR for the identification of viral contamination in river water samples. In contrast, all wastewater samples that were positive in qPCR were also positive by the MBMNA and the detection limit was higher than qPCR; MBMNA was as sensitive as qPCR for viral detection in wastewater samples. Therefore, MBMNA could be a reliable method for the simultaneous detection of viral pathogens, but only in wastewater. For detection of gastrointestinal pathogens xTAG^®^ Gastrointestinal Pathogen Panel - GPP is commercially available [multiplex detection of various viral, bacterial and parasitic nucleic acids in human stool samples (Beckmann et al., 2014; Perry et al., 2014; Wessels et al., 2014; Zboromyrska et al., 2014)]. In comparison to the two previous studies mentioned above only three enteric viruses (norovirus, rotavirus and adenovirus 40/41) can be identified by the GPP (See chapter 4.2).

Viruses such as human papillomaviruses (HPV) are also associated with oncogenesis. HPV belong to those viruses, which require simultaneous detection and typing to identify individual HPV types because the genotype determination is necessary for the investigation of epidemiology and behavior of individual HPV types. Therefore, DDH was designed for detection and genotyping of HPV using L1 consensus (primer systems, which can detect 10 to 100 molecules of HPV targets) resulting in the establishment of a method for simultaneous detection of 26 different HPV genotypes including 18 high-risk HPV and 8 low-risk HPV genotypes (Jiang et al., 2006). Subsequent analysis of the data showed that the 26-plex method precisely discriminated all 18 high-risk HPV targets and also 8 low-risk HPV targets. Another study focused on genotyping HPV also used specific probes targeting a region of the L1 gene (Zubach et al., 2012). DDH was optimized for the detection and genotyping of 46 mucosal HPV types, which are associated with infections of the genital, anal, and oropharyngeal mucosae and the method enables a more comprehensive coverage of HPV types compared with the previously mentioned study, where only 26 types of HPV were genotyped. The DDH was more sensitive than the Linear Array (a leading commercial genotyping method) in terms of distinguishing positive/negative HPV samples, but less sensitive for detection of multiple HPV types; another limitation was the inability of the PCR system to amplify certain variants of HPV68. HPV genotype detection was by combined whole genome amplification and xMAP technology showed that this method is highly specific and sensitive (Lowe et al., 2010). This approach is capable to identify all high risk HPV types with the analytical limit of detection 100 copies plasmid DNA.

Many viruses can cause infections with fatal consequences for human health, e.g., Hendra and Nipah viruses, which can infect cells of the central nervous system and may cause relapsing encephalitis (Clayton et al., 2013), Ebola virus, which causes lethal hemorrhagic disease in humans (Takada and Kawaoka, 2001) or Menangle virus, which causes an influenza-like illness with a rash in humans (Bowden et al., 2012); these zoonotic viruses are linked to bats. The surveillance of zoonotic viruses in wildlife populations is necessary in order to monitor the risk of emerging infectious disease outbreaks. For the complex detection and genotyping of paramyxoviruses in Australian bats two bat virus panel assays (BVPA) for detection of paramyxoviruses in Australian bats (BVPA-1) and for paramyxoviruses and filoviruses in non-Australian bats (BVPA-2) were introduced (Boyd et al., 2015). Examined RNA was extracted from the urine of bats and a total of 532 samples were tested in 11-plex BVPA-1 and 540 field 8-plex BVPA-2; both developed assays were proven to be reliable and accurate.

A number of pathogens, including viruses, are implicated in reproductive diseases of swine. (Chen et al., 2015) combined one-step asymmetric multiplex reverse transcription PCR (RT-PCR) with DDH for simultaneous detection of respiratory syndrome virus (PRRSV), porcine circovirus type 2 (PCV-2), porcine pseudorabies virus (PRV), classical swine fever virus (CSFV), and porcine parvovirus (PPV). All strains of these five viruses were accurately identified. The results showed that the combination of RT-PCR with the DDH assay is more accurate and specific than the other methods, e.g., conventional RT-PCR, and could be a useful tool in the diagnostics of swine diseases. MBMNAs could become very important for veterinary diagnostic testing and (Christopher-Hennings et al., 2013) reported the potential use of MBMNAs for detection of different pathogens in pigs using panels for the multiplex detection of swine pathogens (viruses and bacteria) in serum, lung, oral fluids, feces and spleen or liver.

Although direct diagnosis based on the detection of the nucleic acids of viral pathogens described above prevails, xMAP antibody-based tests for the detection and typing of viruses are also available. MBMI was used to develop a competitive immunoassay that measures HPV type 6, 11, 16, and 18 specific neutralizing antibodies (Opalka et al., 2003); this was later validated for use in epidemiology studies and clinical vaccine trials (Opalka et al., 2003; Dias et al., 2005). MBMI was also compared with a Western blot assay for the detection of HIV-specific antibodies (Kong et al., 2016). The microspheres were coupled with anti-p24 monoclonal antibody and with HIV antigens: gp41, p17, p24, p31, and p66 recombinant protein. The results of both methods showed that MBMI sensitivity was 82.7% and Western blot assay sensitivity was 74.7%. The MBMI was more efficient and precise for screening several parameters and based on the acquired results it was better in HIV diagnostics than Western blots. For the determination of antibodies against HCV in patient serum samples MBMI based on the antigenic properties of four recombinant proteins was designed (Fonseca et al., 2011). Only a small number of samples was tested and that is why the specificity and sensitivity were 100%, but in spite of this the MBMI has the potential to become a viable alternative to standard tests due to its excellent specificity and it may be used for screening of HCV infection. Detection of antibodies against several Epstein-Barr virus (EBV) antigens in nasopharyngeal carcinoma patients (NPC) showed the possibility of simultaneous detection of multiple markers using MBMI, which is not possible with ELISA, and because of the distinct EBV serology spectrum in individual NPC patients, the multiplexed microsphere assay has powerful potential to allow serological diagnosis of NPC in the future (Gu et al., 2008). MBMI showed increased sensitivity and the possibility of quantifying antibodies, antigens, as well as other substances (e.g., hormones, cytokines, tumor markers, etc.), in contrast to conventional ELISA tests (duPont et al., 2005).

### Multiplex Detection and Typing of Bacteria

The majority of applications for multiplex bacterial diagnostics are based on the detection of DNA. The most widely used approaches are based on the DDH, ligation assays or ASPE, but multiplex detection of bacteria may be performed as well using MBMI.

Direct DNA hybridization was used for the detection of pathogens causing foodborne diseases such as acute gastroenteritis and diarrhea, which are usually associated with ingestion of contaminated food. DDH was applied for the typing of 500 *Salmonella* isolates using the genes encoding the flagellar antigens H (*fliC* and *fljB*) (McQuiston et al., 2011). Allele-specific probes for fifteen H antigens, 5 complex major antigens and 16 complex secondary antigens according to the Kauffmann-White serotyping scheme were designed. Comparison of DDH with traditional serotyping methods revealed that the DDH cannot completely replace these methods because unfortunately not all flagellar antigen types were detected. A similar DDH assay for the typing of *Salmonella* focused only on the most common six serogroups of *Salmonella* in the United States (B, C_1_, C_2_, D, E, and O13), as well as serotype Paratyphi A, using the *rfb* genes required for O-antigen biosynthesis in *Salmonella* (Fitzgerald et al., 2007). In contrast with the previous study of McQuiston et al. (2011), the authors showed that the DDH was more specific than traditionally used methods for typing of *Salmonella*.

In the previous sections, it was described how DDH can be used for typing of pathogens; however, in most cases DDH is used only for the detection of pathogens, as described below. (Liu J. et al., 2012) attempted simultaneous detection of the enteric pathogens *Aeromonas*, *Campylobacter jejuni/coli*, *Shigella*, enteroinvasive *Escherichia coli* (EIEC), *Vibrio*, *Yersinia* and as well as *Salmonella* in fecal samples. However, there were some limitations to the method, which included the limited number of clinically significant pathogens or the inability to detect diarrheagenic *E. coli*, protozoa, or viruses. The full capacity of the DDH assay was utilized when the panel was expanded to include the most common bacterial/viral enteropathogens found in stool samples, such as *Salmonella*, *Shigella*, *Vibrio*, toxin B producer *Clostridium difficile*, *Campylobacter*, *Clostridium perfringens*, *Yersinia enterocolitica*, *Aeromonas*, *Escherichia coli* O157:H7, verocytotoxin-producing *Escherichia coli* and adenovirus, Group A rotavirus, norovirus GI and GII and astrovirus (Onori et al., 2014). The results showed that the assay is rapid, sensitive, specific, and reliable for screening and for exploring the etiology of gastrointestinal infections. The sensitivity of MBMNA was demonstrated to be greater than the routine methods (76.3% versus 66.5%), with the exception of *Salmonella* sp. and toxigenic *C. difficile* where the adoption of multiplex PCR did not always result in a significant improvement of specificity. The causative agents were not found in 44 of 245 (18%) of the presumed infectious gastroenteritis cases, but this could be due to the limitations of the detection panel, which did not include allele-specific probes for detection of parasitic enteric pathogens or emerging viruses related to gastroenteritis. Also, using DDH, detection of pathogenic bacteria occurring in environmental samples and causing acute and often fatal diseases (*Bacillus anthracis, Yersinia pestis*, *Francisella tularensis*, and *Brucella melitensis)* was optimized in a multiplexed format to allow the maximum sensitivity and specificity (Wilson et al., 2005). DNA was extracted robotically and in combination with DDH a rapid reliable screening approach was developed. Detection limits were from 100 fg to 10 pg starting DNA concentration when primer sets were multiplexed; in some cases the limits of detection were higher when primer sets were tested separately (range from 10 fg to 10 pg).

Besides the in-house assays developed for multiplex detection of bacteria described above, there are also commercial solutions based on xMAP technology for detection of the most common gastrointestinal pathogens and toxins. The xTAG^®^ Gastrointestinal Pathogen Panel is a multiplex nucleic acid test designed for detection of various bacterial, viral and parasitic nucleic acids in human stool samples (Beckmann et al., 2014; Perry et al., 2014; Wessels et al., 2014; Zboromyrska et al., 2014). The panel allows qualitative detection of *Campylobacter* sp., *Clostridium difficile* (toxin A/B), *Escherichia coli* O157, Enterotoxigenic *E. coli* (ETEC) LT/ST, Shiga-like toxin producing *E. coli* (STEC) *stx1/stx2*, *Salmonella* sp., *Shigella* sp., *Vibrio cholerae*, *Yersinia enterocolitica*, HAdV serotypes 40 and 41, NoV GI and GII, Rotavirus A, *Giardia*, *Cryptosporidium* and *Entamoeba histolytica*. The xTAG GPP was tested and compared with routine tests, which are used in clinical diagnostic laboratories for screening of 17 kinds of enteropathogens, e.g., qRT-PCR kit for detection of viruses, culture methods for detection of bacteria or microscopic examination for detection of parasites (Deng et al., 2015). Samples with discordant results between the routine tests and xTAG GPP were tested by singleplex PCR and sequencing. The overall sensitivity of xTAG GPP was 96.3% and specificity was 99.8%. The sensitivity of xTAG GPP was 100% for all enteropathogens except *Salmonella* sp. (84.9%) and *C. difficile* toxin B (88.6%). The specificity was 100% for all targets except *Salmonella* sp. (99.2%), *Shigella* sp. (99.7%), *C. difficile* toxin B (99.2%), and norovirus GII (98.8%). xTAG GPP is also capable of detecting coinfections; 35 coinfections were detected using xTAG GPP, which is more than by the routine tests. However, the authors also reported some disadvantages as xTAG GPP failed to detect some important diarrheal pathogens (*Aeromonas*, *Plesiomonas shigelloides*) often detected by routine diagnostic tests; further, the detection of *Salmonella* exhibited low sensitivity (84.9%).

Ligation assays are also often used for multiplex detection of pathogenic bacteria. The main advantage over direct hybridization methods is the ability to simultaneously detect diverse signatures such as unique sequences, SNPs, indels and repeats (Song et al., 2010). MOL-PCR was initially optimized for the detection of the biothreat agents *Bacillus anthracis*, *Yersinia pestis*, and *Francisella tularensis* (Deshpande et al., 2010). The pathogen-specific sets of MOLigo pair probes were designed and their specificity and sensitivity were tested using similar species of *Bacillus anthracis*, *Yersinia pestis*, and *Francisella tularensis* and dilutions of isolated DNA, respectively. MOLigo pairs, which showed the highest specificity and sensitivity, were selected for compilation of a final probe panel, which was validated on extracted DNA from infected rodent liver and spleen, human blood or pleural fluid spiked with pathogen DNA. Nine from 10 unknown samples were successfully identified using the final probe panel. The results also showed the ability of this method to simultaneously detect multiple different signatures (SNPs, indels and repeats). The versatility of MOL-PCR was utilized when simultaneous detection of *Bacillus anthracis*, *Yersinia pestis*, and *Francisella tularensis* was supplemented by characterization of antibiotic resistance (ciprofloxacin and doxycycline) of these bacteria based on SNP analysis (Song et al., 2010). The allele-specific probes for detection and characterization of all the known resistance determinants performed well when tested individually, but multiplex use did not provide satisfactory results. Due to the ability to simultaneously detect diverse signatures such as unique sequences, SNPs, indels, and repeats, MOL-PCR can be used as a genotyping method as described below. A MOL-PCR-based 8-plex SNP typing method for *Mycobacterium tuberculosis* complex (MTBC) based on two phylogenetically equivalent sets of SNP markers that are specific for the six main human-associated lineages of MTBC was introduced (Stucki et al., 2012). MOL-PCR was compared with TaqMan qPCR and the obtained results showed that the sensitivity and specificity of both methods were similar (specificity 100%, sensitivity 98.6% MOL-PCR, 98.8% TaqMan) and that both methods were of comparative cost. MOL-PCR was ideal for classification of unknown isolates, while TaqMan qPCR was faster for confirmation of unknown isolates. MOL-PCR was also successfully used for genotyping of *Bacillus anthracis* in a 13-plex assay to score 13 phylogenetically lineage-specific canonical SNPs within the genome of *Bacillus anthracis* (Thierry et al., 2013).

Allele-specific primer extension was applied for identification of bacteria (Lin et al., 2008) even though it is more commonly used for SNP genotyping. ASPE was used for the identification of *Acinetobacter sp.* and antimicrobial susceptibilities of the clinical *Acinetobacter* species isolates were also determined (Lin et al., 2008). The 16S-23S rRNA gene intergenic spacer (ITS) regions of 13 distinct *Acinetobacter* species were amplified and then multiplex ASPE was performed. It was shown that this multiplex identification of *Acinetobacter* sp. is applicable also for determination of antibiotic resistance of the clinical *Acinetobacter* isolates. ASPE was compared with SBCE for identification of bacterial samples (Ye et al., 2001) and both methods provided similar results as they managed to correctly classify 17 bacterial species into 17 groups.

In addition to MBMNA also MBMI can be used for the direct multiplex detection of bacteria and their products (Dunbar et al., 2003). In MBMI direct fluorescence (detection antibody that incorporates a fluorescent label) is used for detection of reaction or of emerging product in contrast to ELISA and, in addition, MBMI enables measurement of multiple analytes simultaneously. For this reason, MBMI is preferred because time for detection is reduced and also test sensitivity is increased (Jun et al., 2012). Capture sandwich immunoassays (CS) were successfully applied for detection of organism-specific antibodies using microspheres coupled with antibodies for *Salmonella*, *Campylobacter*, *Escherichia coli*, and *Listeria* and it has been demonstrated that MBMI is a suitable method for multiplex detection of bacteria occurring in foodstuffs (Kim et al., 2010) or for detection of *Brucella* sp. from milk using capture-sensitive monoclonal antibodies for the lipopolysaccharide (LPS) O-antigen of *Brucella* sp. (Silbereisen et al., 2015). MBMI was also applied to test bacterial contamination of foods through the detection of staphylococcal enterotoxin B (SEB) (Kim et al., 2010), staphylococcal toxin A (SEA), and toxic shock syndrome toxin (TSST) produced by various strains of *Staphylococcus aureus* (Simonova et al., 2014) using sandwich immunoassays in which microspheres were conjugated with specific antibodies. A similar approach was used for the detection of pneumococcal serotype-specific polysaccharide and C-polysaccharide (C-Ps) antigens from urine samples (Sheppard et al., 2011). For the detection, MBMI was combined with the Binax NOW *Streptococcus pneumoniae* antigen detection kit. The specificity of MBMI was determined by testing 85 serotypes of *S. pneumoniae* and other strains of streptococci; 18 of the 26 non-pneumococcal serotypes gave C-P positive results, which showed that MBMI could be used for diagnosis of infection caused by *S. pneumoniae* only in combination with the Binax NOW assay.

### Multiplex Detection of Parasitic Agents

Parasitic zoonoses are recorded worldwide and some of them have endemic character. Parasitic agents may pass from animals to humans in several ways, e.g., by direct contact, vector, consumption of raw or undercooked foodstuffs containing the infective stages or by infective stages released into environment (Hubalek, 2003). In the context of animal health and human food consumption, a list of the top ten parasites has been defined by the UN’s Food and Agriculture Organization (FAO) and World Health Organization (WHO) (**Table 3**). Although in the last decades a number of novel diagnostic methodological approaches has been developed, the current diagnosis of some parasitic diseases is still based only on a combination of clinical signs, anamnesis, and direct visual identification of parasitological objects (Anderson et al., 2015). The most common conventional diagnostic methods, such as microscopic examination, biochemical assays or ELISA, are available, but they are laborious, time-consuming and in many cases not reliable (Navidad et al., 2013). Improvements in this field are represented by molecular methods, including also routine PCR diagnostics, increasingly used for detection mainly of intestinal parasites, which are easy to recover from fecal specimens (Taniuchi et al., 2011) or potentially useful for other parasites found in secretions. With regard to the fact that parasites might exhibit very strictly confined localization within the host’s body – intracellular/extracellular or tissue/organ, sampling can be very problematic and it often leads to a false negative results.

**Table 3:** Foodborne parasites with the greatest global impact (Anonymous, 2014).

Parasite	Type	Occurrence
*Taenia solium*	Tapeworm	Pork
*Echinococcus granulosus*	Hydatid worm or dog tapeworm	In fresh produce
*Echinococcus multilocularis*	Tapeworm	In fresh produce
*Toxoplasma gondii*	Protozoa	In meat from small ruminants, pork, beef, game meat (red meat and organs)
*Cryptosporidium* sp.	Protozoa	In fresh produce, fruit juice, milk
*Entamoeba histolytica*	Protozoa	In fresh produce
*Trichinella spiralis*	Worm	Pork
*Opisthorchiidae*	Flatworm	in fresh water fish
*Ascaris* sp.	Roundworm	In fresh produce
*Trypanosoma cruzi*	Protozoa	In fruit juice


Outbreaks of diarrheal diseases are caused by a wide range of pathogens, including parasites. Stool microscopy (detection of eggs, parts of bodies etc.) is the gold standard in the diagnostics of intestinal parasites. However, the presence of parasites in stool may vary and could be naturally low, requiring multiple sampling. In fact, up to 80% of all cases of diarrhea remain without confirmed etiology (Vernacchio et al., 2006). Therefore, there is space for the development of more sensitive diagnostic assays (Taniuchi et al., 2011), which should provide more precise determination. Among the modern molecular diagnostic methods qPCR assays are most frequently used for determination of intestinal parasites. In areas where co-infections are common (up to 22% of cases are caused by two or more pathogens) (Jansen et al., 2008; Friesema et al., 2012), the application of multiplex assays is of great benefit. Several pioneering works have been published in relation to this topic. To date, in parasitology, improved multiplex qPCR assays were adapted to DDH, which enables parallel diagnosis of seven intestinal parasites (Taniuchi et al., 2011); separate reactions were optimized – 3-plex for protozoa (*Cryptosporidium* sp., *Giardia intestinalis*, and *Entamoeba histolytica*) and 4-plex for helminths (*Ancylostoma duodenale, Ascaris lumbricoides, Necator americanus*, and *Strongyloides stercoralis*). The final calculated sensitivity was 83% and specificity was 100%. The results of both DDH assays were equivalent or better in comparison to the parent multiplex qPCR. Moreover, this approach has been developed as a commercial diagnostic xTAG GPP tool– a 19-plex assay, which enables *inter alia* detection of the protozoa *G. intestinalis, E. histolytica* and *Cryptosporidium* sp. The overall performance of xTAG GPP compared with conventional methods (standard culture, microscopic examination, immunochromatographic tests, qPCR) showed a sensitivity of 94.5% (range 90 to 97%) and a specificity of 99% (range 98,5% to 99,9%) (Claas et al., 2013; Mengelle et al., 2013; Navidad et al., 2013). If multiplexing more than 20 targets, the limit of detection might be reduced for individual targets when compared to single-target detection (Navidad et al., 2013). However, the identification of multiple pathogens revealed that very often (in up to 65% of samples), the physicians do not request testing for the proper pathogen (Claas et al., 2013). Therefore, multiplexing refines the diagnosis and contributes to the selection of a suitable treatment.

It was mentioned above that microsphere-based assays can be arranged also as multiplex indirect immunoassays, although the conventional singleplex ELISA still represents the gold standard in serodiagnostics for screening of individual human/animal or higher numbers of samples at a population level (Ruitenberg et al., 1983; Nockler et al., 2000; Dubey et al., 2005). Recently, some studies have been done in order to improve the potential of this serological method and to upgrade it to the multiplex level. These studies are mostly focused on parasites with the ability to migrate through the tissues of the host’s body – where PCR based detection would not be reliable. In this context, the most studied group of parasites are representatives from the phylum Nematoda, including also the important human pathogens, the *Trichinella* sp. The larvae may infect humans during the ingestion of raw or undercooked meat, mainly pork (domestic pig, wild boar) and can induce disease, whose consequences can be fatal (Dupouy-Camet, 2000; Pozio and Murrell, 2006). Inspection of meat for the most important species, *Trichinella spiralis*, is mandatory at slaughter (Anonymous, 2015), but currently used methods like artificial digestion and microscopic examination of pooled meat samples (Nockler et al., 2000) are archaic and usually do not properly reflect the real infection. Therefore, serodiagnostic methods are considered as a possible alternative and xMAP technology in the form of ISA, using excretory/secretory (E/S) products, was also developed and tested. The effectivity of ISA was tested with *T. spiralis*-positive pig meat samples. The system was developed as a duplex assay (with *Toxoplasma gondii*), using goat anti-swine secondary antibodies against specific antibodies. The results of this study corresponded to the infection status of the animals with an assay sensitivity of 68% and specificity of 100% (Bokken et al., 2012). When the immunoglobulin binding protein A/G (generic Ig-binding protein), which can be used in multiple species in contrast with goat anti-swine secondary antibody, was included, the results showed a similar specificity of 95%, but an increase in sensitivity from 88% for anti-swine antibody to 94% with protein A/G. The xMAP technology-ISA exhibited 87% sensitivity and 95% specificity in comparison with the commercial Pourquier ELISA, and 98% sensitivity and 95% specificity in comparison with the Safepath ELISA (van der Wal et al., 2013).

With the rising popularity of MBMIs, ISA was also developed for other members of Nematodes, such as representatives from the genus *Toxocara* (Anderson et al., 2015). The infection by these parasites is typically peroral at areas contaminated by embryonated roundworm eggs, e.g., sand from childrens’ playgrounds. The recombinant *T.canis* and *T. cati* E/S antigens Tc-CTL-1 and Tc-TES-26 were used to detect toxocara-specific antibodies in sera from humans pre-diagnosed as positive for visceral and ocular larval migrans (VLM, OLM). The specificity of ISA was 94% for both sets of samples, but there were differences in the sensitivity, which was 99% for VLM and 64% for OLM samples. It was recorded that a combination of recombinant antigens improves sensitivity in comparison with conventional immunoassays (e.g., Western Blot, ELISA), which employ native E/S antigens isolated from larvae (limited availability) that also exhibit cross-reactivity with antibodies from other helminthic infections so reducing its usefulness in regions with poly-parasitism.

Within the unicellular parasitic protozoa ISA was tested in representatives from the genus *Toxoplasma.* Unlike *T. spiralis*, no such regulations for meat control exist for *T. gondii*, even though its prevalence is higher and health complications can be very severe. Recombinant tachyzoite surface protein (SAG-1) was used for simultaneous serological detection in a set with *T. spiralis* E/S (Bokken et al., 2012). Similarly to *T. spiralis*, the results exactly reflected the load of infection; sensitivity was 86% and specificity was 96% for *T. gondii.* The obtained results repeatedly underline the potential of these assays for further implementation in routine diagnostic screening of a wide range of parasites.

As we have descibed, the ISA represents an improved methodological alternative to current serological diagnostics, enabling multiplex detection of pathogenic agents with higher sensitivity.

### Multiplex Detection and Typing of Fungal Pathogens

Traditional diagnostic methods for the identifications of fungal pathogens are mostly based on phenotype analysis of fungal cultures or detection of antigens (polysaccharides), but these approaches are time-consuming and not very accurate (Diaz and Fell, 2004; Bovers et al., 2007; Landlinger et al., 2009; Babady et al., 2011). Rapid and correct identification methods are important for efficient therapy (Diaz and Fell, 2004), however, available qPCR assays have various levels of sensitivity and specificity and often have a limited range, targeting only a few yeasts or mold species (Landlinger et al., 2009; Babady et al., 2011).

The need for rapid and correct identifications of fungal pathogens was addressed by development of xMAP technology based detection methods (Diaz and Fell, 2004; Page and Kurtzman, 2005; Das et al., 2006; Bovers et al., 2007; Babady et al., 2011; Balada-Llasat et al., 2012; Farooqi et al., 2012; Landlinger et al., 2009). Majority of xMAP applications for the multiplex detection and identification of fungal pathogens are based on the capture of fungal nucleic acid by DDH assays.

To perform rapid and accurate identifications of fungal pathogens in immunocompromised individuals, the DDH was designed detect a wide range of the most commonly occurring clinically relevant fungal pathogens including species of the genera *Aspergillus* and *Candida* and other important pathogens such as *Cryptococcus*, *Fusarium*, *Trichosporon*, *Mucor*, *Rhizopus*, *Penicillium*, *Absidia*, and *Acremonium* (Landlinger et al., 2009). The DDH was used mainly for identifications of fungi due to its ability to detect coinfections with multiple fungal species in patients and may contribute to improved diagnosis of invasive fungal infections.

Studies employing xMAP technology were developed and successfully used to identify individual fungal species within *Candida* sp. (Page and Kurtzman, 2005; Farooqi et al., 2012), or *Trichosporon* sp. (Diaz and Fell, 2004). In these studies, DDH assays for fast and accurate detection and identification of important fungal pathogens were developed. In another study, the xMAP technology was used for genotyping of human pathogenic *Fusarium* sp. (O’Donnell et al., 2007). Fusaria were genotyped also by sequence analysis. The independent comparison of the results obtained via xMAP technology with results obtained via sequencing showed the xMAP incorrectly identified some of *Fusarium* isolates.

Besides the in-house assays described above, commercial kits have also been developed for the detection of fungal pathogens by xMAP, e.g., xTAG^®^ Fungal Analyte-Specific Reagents (ASR) assay and the sensitivity and specificity of the assay were tested within identification of fungal isolates and positive blood culture bottles (Babady et al., 2011). The *Candida* 7-plex assay was tested within 43 of *Candida* strains and 16 bacterial strains with no-cross-reaction with any of the bacterial strains. The sensitivity and specificity were 100%. Using 11-plex assay were tested 51 mold species and the assay correctly identified all species of *Aspergillus*, with 100% specificity and sensitivity except *A. niger* (0/8 isolates). Other molds were identify also with 100% specificity and sensitivity except *Mucor* (0/6 isolates) and *Rhizopus* (1/6 isolates). Besides the testing of fungal isolates also positive blood culture bottles were tested for the presence of *Candida* species using *Candida* 7-plex assay. The sensitivity and specificity of the assay was 100% for each species. The mold 11-plex assay did not detect one *Rhizopus* species and the *A. niger* strains, so the results were similar as the previous mentioned results in the course of identification of fungal isolates.

In addition, ASR for identification of *Candida* species do not distinguish between members of *Candida* complexes, e.g., phenotypically indistinguishable groups II and III of *C. parapsilosis* (group I), which have been renamed *Candida orthopsilosis* and *C. metapsilosis*. Similarly, ASR for identification of *A. fumigatus* were unable to distinguish between members of the *A. fumigatus* complex. The results showed that xTAG^®^ Fungal ASR assay could be used as an adjunct to culture. The mold 11-plex assay has been developed specifically for the detection of specific species of mold, which may be reason why *Rhizopus, Mucor*, and *A. niger* have not been identified. Due to the equal treatment of infections caused by genera *Mucor* and *Rhizopus*, it would be better to design a panel to detect the most common genera of fungi, and not to focus on the detection of particular species.

The results showed that the xTAG^®^ Fungal ASR assay is an attractive alternative to reference methods, due to its speed and ability to simultaneously identify multiple fungal species (Balada-Llasat et al., 2012).

DDH assay is able to not only identify the fungal pathogens, but it can be used for a genotyping of fungal pathogens. It was applied for identification of closely related pathogenic yeasts *Cryptococcus neoformans* and *Cryptococcus gattii* that may cause meningoencephalitis in immunocompromised individuals (Bovers et al., 2007). Six haploid genotypic groups within these pathogens can be distinguished by several molecular methods e.g. PCR fingerprinting or intergenic spacer genotyping. Besides these haploid groups, hybrids have been described as well. AD hybrids are hybrids between the two varieties of *C. neoformans* and also hybrids between *C. neoformans* var. *neoformans* and *C. gattii* have been described. The DDH has been adapted for the detection of the genotypes within *Cryptococcus neoformans* and *Cryptococcus gattii*. The detection limit was calculated from 4 × 10^1^ to 2 × 10^3^ cells for the various specific probes for each of the six haploid genotypic groups. The results showed that DDH is highly specific method and it is possible not only identify cryptococcal isolates at the species and genotype levels but also allows identification of hybrid isolates that have two alleles of the specific probes region and also able to identify cryptococci in cerebrospinal fluid. However, the optimization of DNA extraction methods is needed before routine use in clinical laboratories.

## Conclusion

Detection and identification of pathogens, as well as an understanding of pathogen variation, the pathogenesis of the diseases they cause, and timelines of infection and antimicrobial resistance, are all required in order to obtain the full picture of disease progression and to select effective cures for infected individuals or populations. As the amount of input data required for such decisions increases, so too does the number of tests that are required during laboratory examinations. The multiplex assays for the detection and typing of pathogens using xMAP technology are tools of choice as they are capable of providing all of the important information within a reasonable timeframe, and without excessive labor or costs. The major improvement of xMAP assays is that they add another dimension to the simple detection, which is represented by the simultaneous analysis of many targets within a single sample, and they therefore represent complementary tools to procedures for the detection and quantification of pathogens such as qPCR, culture, or ELISA assays. The significance of such a complex approach for the multiplex detection has grown in recent years, which is documented by the increase in published data and of application of the commercial assays in routine diagnostics.

## Author Contributions

Conception of the review: PK; Design of the work: VM, NR, PK; Writing this review: NR and VM (These authors contributed to this work equally); Revision of the manuscript: MK, PM, PK; All authors approved the version to be published in *Frontiers in Microbiology* and agreed to be accountable for all aspects of the work.

## Conflict of Interest Statement

The authors declare that the research was conducted in the absence of any commercial or financial relationships that could be construed as a potential conflict of interest.
